# The association between serum C-reactive protein and macronutrients and antioxidants intake in hemodialysis patients

**Published:** 2015

**Authors:** A Kooshki, E Samadipour, R Akbarzadeh

**Affiliations:** * Department of Nutrition & Biochemistry, Sabzevar University of Medical Sciences, Sabzevar, Iran; ** Para Medicine School, Sabzevar University of Medical Sciences, Sabzevar, Iran

**Keywords:** serum C-reactive protein, macronutrients, antioxidants, cardiovascular, hemodialysis

## Abstract

**Background:**Despite the high levels of inflammation in hemodialysis patients and the effects of diet on systemic inflammation, such as the development of atherosclerosis and cardiovascular disease, few studies have evaluated the relationship of macronutrients and antioxidants intake with serum C-reactive protein (CRP). Therefore, this study assessed the relationship between serum high sensitivity CRP (hs-CRP) with macronutrients and antioxidants intake and serum albumin.

**Methods:**This cross-sectional study used census sampling to select 75 hemodialysis patients (35 men and 40 women) who attended the hemodialysis department of Vaseie Hospital of Sabzevar, Iran. After obtaining the written consent, all the patients were interviewed and dietary data was collected by using a semi-quantitative food frequency questionnaire including 160 food items. Diet analysis was performed with Nutritionist IV. Before being connected to the dialysis machine, 5 cc fasting blood samples were obtained from all participants and serum hs-CRP and albumin levels were measured. All the statistical analyses were conducted with SPSS -for Windows, version 16.0.

**Results:**The patients’ mean body mass index was 20.09 ± 3.27 kg/ m2. The participants’ intake of antioxidants and all macronutrients, except for carbohydrates and proteins, was less than the standard levels. Moreover, the hs-CRP had significant inverse relationships with serum albumin (P=0.0001) and vitamin E and C intakes but was not significant. Also, a significant relationship was observed between hs-CRP levels and the intake of energy (P=0.002) and protein (P=0.0001).

**Conclusion:**Our findings indicated hs-CRP levels of hemodialysis patients to have significant inverse relationships with serum albumin and vitamin E and C intakes but was not significant. Also, a significant relationship was observed between hs-CRP levels and the intake of energy and protein.

## Introduction

The number of patients on hemodialysis, a treatment option for chronic renal failure, is increasing constantly and rapidly [**1**]. Over 11,000 patients are currently undergoing hemodialysis in Iran and this number increases with about 8% every year. The mortality rate of dialysis patients is 4.0-5.3 times higher than that of the general population. Besides, cardiovascular diseases are responsible for 40%-50% of the deaths among dialysis patients and cause a mortality rate 5-20 times higher than that in the general population [**2**]. Even the presence of classical risk factors of cardiovascular diseases in these patients cannot justify such a high mortality rate. In fact, it is now evident that systemic inflammation plays an important role in the development of atherosclerosis [**3**].

The prevalence of inflammation has been reported as 35%-65% among hemodialysis patients [**3**,**4**]. Although the exact reasons of inflammation in hemodialysis patients are yet to be clarified [**5**], C-reactive protein (CRP), a positive acute phase protein produced in the liver, is an inflammatory biomarker whose levels increase in response to inflammation [**6**,**7**]. Studies have suggested high serum CRP as a strong predictor of death, especially due to cardiovascular disease, in hemodialysis patients [**8**,**9**] and have reported such high levels in 30%-50% of the patients [**10**-**12**]. A low concentration of serum albumin has also been identified as a strong predictor of death due to cardiovascular diseases in patients with chronic renal failure [**13**], i.e. every 1 g/ dL decrease in serum albumin increases the patients’ risk of mortality by seven times [**14**]. Moreover, every 10-unit increase in the dialysis malnutrition score (DMS) and malnutrition inflammation score (MIS) has been found to increase the risk of mortality by 7.7 and 10.0 times, respectively [**14**].

Several factors such as anorexia, limited intake of some food groups, loss of water-soluble nutrients such as vitamin C during hemodialysis, accumulation of nitrogen compounds in blood and uremia, reduced neuropeptide Y, increased serum leptin, and catabolic state due to increased inflammatory cytokines (e.g. CRP) may cause undesirable nutritional status in hemodialysis patients [**15**-**17**]. In addition, the high level of oxidative stress in these patients aggravates inflammation [**18**] and increases the need for antioxidants. Despite the crucial effects of diet on systemic inflammation, few studies have evaluated the relationship between the intake of macronutrients and antioxidants and serum CRP. Therefore, this study was conducted to investigate the association between serum high-sensitivity CRP (hs-CRP) and macronutrients and antioxidants intakes in hemodialysis patients.

## Materials and Methods

This cross-sectional study used census sampling to include 75 hemodialysis patients (35 men and 40 women) who were referred to the Hemodialysis Department, Vaseie Hospital, Sabzevar (Iran). After obtaining a written consent from all participants, they were privately and individually interviewed by a trained and experienced questioner.

After dialysis, the patients’ weight and height were measured with a Seca digital scale and a tape measure, respectively. All measurements were performed by a particular person and according to the standard instructions while the subjects were wearing light clothing and no shoes. The weight and heights were recorded with accuracy of 100 g and 1 cm, respectively. The body mass index (BMI) was calculated as weight in kilograms divided by height in squared meters. Moreover, the necessary data on dietary intake were obtained through a semi-quantitative food frequency questionnaire including 160 food items. Dietary intake analysis was performed with Nutritionist IV (Axxya Systems, USA).

Patient files were used to record the adequacy of dialysis, which was quantified as Kt/ V (where K, t, and V are dialyzer clearance of urea, dialysis time, and volume of distribution of urea, respectively) [**19**]. Before being connected to the dialysis machine, 5 cc fasting blood samples were collected from all participants. The samples were centrifuged at 2500 rounds per minute for five minutes. The separated serums were then stored in the freezer until biochemical tests. Serum albumin concentration was measured by using bromocresol green kits (Pars Azmoon Co., Iran). Hs-CRP concentrations were measured with enzyme-linked immunosorbent assay (ELISA) kits (Diagnostics Biochem Canada Inc, Canada).

Statistical analyses of data were performed by using SPSS for Windows, version 16.0 (SPSS Inc., Chicago, IL, USA). The mean and standard deviation of quantitative variables were calculated. Pearson’s correlation analysis was employed to investigate the correlations between continuous quantitative variables.

## Results

The mean BMI of the patients was 20.09 ± 3.27 kg/ m2. The demographic characteristics of the patients are shown in **Table 1**.

**Table 1:** Mean and standard deviation of investigated indicators in hemodialysis patients

Index	Mean
Age (year)	51.81 ± 16.06
Weight (kg)	55.52 ± 10.63
(kt/ v) adequacy of dialysis	1.7 ± 0.9
Dialysis time (hour)	4 ± 0.5
Duration of dialysis (months)	21 ± 20
Serum Albumin (gr/ dl)	4.64 ± 0.3
CRP (mg/ L)	4.88 ± 2.48

**Table 2** compares the patients’ energy, macronutrients, and antioxidants intake with standard levels. As it was seen, the participants’ intake of antioxidants and all macronutrients, except for carbohydrates and proteins, was lower than the standard levels.

**Table 2:** Mean and standard deviation of energy, macronutrients, and antioxidants intake in hemodialysis patients

Nutrients	Mean ± SD	DRI`
Energy (Kcal/ d)	1803.5 ± 415.76	< 60 years: 35 Kcal/Kg/d ≥ 60 years:30-35 Kcal/Kg/d
Carbohydrate (gr/ d)	286.44 ± 70.92	50-55% Kcal/ d
protein (gr/ d)	69.30 ± 21.41	1.2 gr/ d≤
Lipid (gr/ d)	33.16 ± 16.88	25-30% Kcal/ d
SFA (gr/ d)	9.02 ± 5.04	< 10% Kcal/ d
MUFA (gr/ d)	11.22 ± 7.32	15% Kcal/ d
PUFA (gr/ d)	7.61 ± 5.27	< 10% Kcal/ d
Cholesterol (mg/ d)	144.01 ± 102.62	300 mg/ d
Fiber (gr/ d)	2.10 ± 1.3	20-25 gr/ d
Vitamin A (mcg)	369.62 ± 617.13	4000-5000
Vitamin E(mg/ d)	1.14 ± 0.51	15
Vitamin C(mg/ d)	53.17 ± 44.07	70-80
Selenium (mcg/ d)	0.50 ± 0.028	50-55

The relationship of serum hs-CRP with serum albumin and macronutrients and antioxidants intake showed that hs-CRP had significant inverse relationships with serum albumin (P0.0001) and vitamin E and C intakes but was not significant. Also, a significant relationship was observed between hs-CRP levels and intake of energy (P=0.002) and protein (P=0.0001).

## Discussion

According to our findings, a significant inverse relationship was observed between serum hs-CRP and serum albumin. Studies have shown that decreased serum albumin can strongly predict mortality, especially mortality due to cardiovascular diseases, in these patients [**20**]. Albumin is also an important indicator of nutritional status of hemodialysis patients and a negative acute phase reactant [**21**,**22**]. Kaysen reported approximately similar results and found 7% of the patients to have serum albumin levels lower than normal [**23**]. Research has suggested that the risk of mortality begins when serum albumin concentrations fall to as low as 4 g/ dl and increases considerably with levels below 3 g/ dl [**24**].

The inverse relationship between serum hs-CRP (positive acute phase protein) and serum albumin was also confirmed by Qureshi [**25**]. However, Nasri, did not observe any significant relationship between serum CRP and serum albumin [**26**]. In an effort to establish relationships between the dietary intake of antioxidants and serum hs-CRP concentrations, we found vitamin E and C intakes to have an inverse relationship with serum hs-CRP. Naghashpour and Bertran reported consistent results as they showed significantly lower vitamin A and β-carotene intake (precursor of vitamin A) in people with higher levels of serum hs-CRP compared to those with lower serum hs-CRP [**27**,**28**]. Similarly, Fredrikson et al. did not detect any significant relationship between serum hs-CRP and vitamin E intake [**29**]. Likewise, Naghashpour found no relationship between serum hs-CRP and vitamin C and selenium intake [**28**]. In contrast, Kafshani could establish a significant inverse relationship between selenium intake and serum CRP [**30**].

Antioxidants affect plasma CRP probably through their effect on upstream cytokines, especially tumor necrosis factor alpha (TNF-α), interleukin 1β (IL-1β) and interleukin-6 (IL-6), that are the main producers of acute phase response [**31**]. Researchers believe that vitamin C inhibits lipopolysaccharide activity, which causes the production of TNF-α and IL-6. It also inhibits the production of interleukin-2 (IL-2) after the incidence of stressful factors. Several mechanisms have been suggested for oxidative and non-oxidative processes [**32**]. Accordingly, oxidative damage leads to an inappropriate activation of nuclear transcription factor, leading to increased expression of inflammatory proteins. Antioxidants such as vitamin C inhibit the activation of this pathway and substantially reduce plasma F2-isoprostane (an oxidative stress marker) levels [**33**]. Vitamin E has also been shown to decrease the activity of lipoxygenases, which inhibit the activity of IL-1β. As CRP production is regulated directly by IL-6 and IL-1β, the down-regulation of these factors by antioxidants would reduce serum CRP [**34**,**35**].

In the current study, a significant relationship was observed between hs-CRP levels and the intake of energy and protein. Consistent results were reported by Naghashpour who found no significant relationship between the received energy and hs-CRP levels. However, he did not assess the relationship between serum hs-CRP and macronutrients [**28**]. Moreover, Kafshani indicated the absence of relationships between received macronutrients and CRP levels [**30**]. In contrast, Bertran et al. showed that lower intake of many nutrients such as carbohydrate, protein, lipid, and tocopherol was associated with higher concentrations of plasma CRP [**27**]. These inconsistencies may be due to the differences in the software used in food analysis, measurement methods, and sample size.

## Conclusion

This research revealed that hemodialysis patients’ intake of antioxidants and all macronutrients, except for carbohydrates and protein, is lower than the standard levels. It also indicated significant inverse relationships only between hs-CRP receiving and serum albumin and vitamin E, C and selenium intakes but was not significant. Also, a significant relationship was observed between hs-CRP levels and the intake of energy and protein.

**Acknowledgement**

The authors appreciate the respected Vice Presidents of Research and Education, the head and dialysis department staff of Vaseie Hospital, Sabzevar University of Medical Sciences, and all patients who kindly participated in this study.
